# Synergistic Effects of Silica Nanoparticles, Chitosan and *Bacillus velezensis* AAHM-BV2301 on the Growth, Immunity, Gut Microbiota and Disease Resistance of Asian Seabass (*Lates calcarifer*)

**DOI:** 10.3390/biom16010088

**Published:** 2026-01-05

**Authors:** Jasper Kit Tangal, Anurak Uchuwittayakul, Kriengkrai Satapornvanit, Prapansak Srisapoome

**Affiliations:** 1 Laboratory of Aquatic Animal Health Management, Department of Aquaculture, Faculty of Fisheries, Kasetsart University, 50 Paholayothin Rd., Ladyao, Chatuchak, Bangkok 10900, Thailand; jasperkitvillar.t@ku.th (J.K.T.); ffisarb@ku.ac.th (A.U.); 2 Center of Excellence in Aquatic Animal Health Management, Faculty of Fisheries, Kasetsart University, 50 Paholayothin Rd., Ladyao, Chatuchak, Bangkok 10900, Thailand; 3 Department of Fishery Biology, Faculty of Fisheries, Kasetsart University, 50 Paholayothin Rd., Ladyao, Chatuchak, Bangkok 10900, Thailand; ffiskks@ku.ac.th

**Keywords:** physicochemical nanoscaffold, nanoparticle-probiotic interactions, innate immunity, microbiome modulation, *Cetobacterium somerae*, *Vibrio vulnificus*

## Abstract

In this study, the synergistic effects of dietary *Bacillus velezensis* AAHM-BV2301, silica nanoparticles (SiNPs), and chitosan (CS) on the growth performance, innate immunity, gut microbiota, and disease resistance of Asian seabass (*Lates calcarifer*) fingerlings were evaluated. A total of 400 fish (11.25 ± 2.12 g) were assigned to five dietary treatments for 30 days: control, BV (1 × 10^8^ CFU/kg feed), BVSiNP (1 × 10^8^ CFU/kg + 2 mg SiNP/kg), BVCS (1 × 10^8^ CFU/kg + 15 g CS/kg), and BVSiNPCS (combined additives at the same concentrations). The growth indices (WG, SGR, RGR, and FCR) significantly increased in the fish fed BVSiNPs, whereas the level of innate immunity increased across all the supplemented groups, with BVCS and BVSiNPCS having the strongest respiratory burst and lysozyme activities. The tissue-specific modulation of immune-related genes (*α2M, HSP70*, *Mx*, and *C3*) was most pronounced in BVSiNP-fed fish, particularly in the gills and liver. Gut microbiome profiling revealed enrichment of *Cetobacterium somerae* in response to BV-based treatments, whereas BVSiNPCS induced the greatest increase in microbial richness and network connectivity. Postchallenge survival against *Vibrio vulnificus* was significantly greater in the BV and BVSiNP groups (*p* < 0.05). Overall, SiNPs acted as functional enhancers of the *B. velezensis* probiotic, supporting improved growth, immune activation, and microbiota restructuring. These results highlight the potential of nanoparticle-integrated synbiotics for microbiome-targeted health management in aquaculture.

## 1. Introduction

Asian seabass (*Lates calcarifer*) is a commercially significant euryhaline teleost widely cultivated across the Asia-Pacific region, with Thailand accounting for approximately 43% of global production between 2000 and 2020 [1,2,3,4,5]. However, the intensification of culture systems—particularly the expansion into low-salinity earthen ponds—has exacerbated the prevalence of infectious diseases, such as vibriosis, particularly *Vibrio vulnificus*, posing a major constraint on sustainability [3,6]. While antibiotics are commonly used to manage these outbreaks, increasing concerns regarding antimicrobial resistance (AMR), drug residues, and environmental dysbiosis have necessitated the development of nonantibiotic functional feed strategies [7,8,9,10,11].

To address these challenges, functional additives such as probiotics and prebiotics have gained prominence. *Bacillus velezensis*, a spore-forming bacterium capable of producing extracellular enzymes and antimicrobial metabolites, has shown promise as an immunomodulator in aquaculture [12,13,14,15]. Concurrently, the ability of chitosan, a polysaccharide derived from chitin, to stimulate innate immunity, enhance mucosal protection, and selectively enrich beneficial gut taxa is well documented [16,17,18]. Despite their individual benefits, the efficacy of conventional synbiotics (probiotic–prebiotic combinations) is often limited by gastrointestinal survival and the stability of bioactive metabolites [9,10,11].

To overcome these limitations, this study integrates silica nanoparticles (SiNPs) as a novel physicochemical scaffold. SiNPs are biocompatible nanomaterials with a high surface-area-to-volume ratio that increase nutrient digestibility and can modulate microbial activity in the gut [19,20,21]. We hypothesize that SiNPs function as physicochemical nanoscaffolds. By increasing the specific surface area and modifying the interfacial properties of the diet, this scaffold enhances the mucosal residence time and bioavailability of the antimicrobial peptides and enzymes secreted by *B. velezensis*, thereby optimizing the therapeutic efficacy of the synbiotic system in the Asian seabass gut.

The gut microbiota plays a pivotal role in mediating these effects, influencing host immune development, nutrient metabolism, and disease resistance [22,23,24]. Recent advances in high-throughput full-length 16S rRNA sequencing have enabled detailed characterization of microbial shifts in response to dietary modulation. Therefore, the aim of this study was to evaluate the synergistic effects of dietary supplementation with *B. velezensis* AAHM-BV2301, chitosan, and SiNPs on the growth performance, innate immunity, and gut microbiota architecture of Asian seabass fingerlings. Furthermore, we investigated the protective efficacy of this formulation against *V. vulnificus* infection to provide a mechanistic basis for its use in sustainable, antibiotic-free aquaculture.

## 2. Materials and Methods

### 2.1. Ethical Approval and Animal Welfare Compliance

The experiments were conducted in compliance with the Ethical Principles and Guidelines for the Use of Animals established by the National Research Council of Thailand and were approved by the Animal Ethics Committee of Kasetsart University, Bangkok, Thailand (approval code ACKU68-FIS-014). The research protocol strictly followed ethical guidelines for the use of animals in scientific research, ensuring the welfare and humane treatment of the fish throughout the experiment.

### 2.2. Probiotic Culture Preparation

*Bacillus velezensis* AAHM-BV2301, which was originally isolated from the intestine of healthy Asian seabass and maintained at the Center of Excellence in Aquatic Animal Health Management (CE AAHM), Department of Aquaculture, Faculty of Fisheries, Kasetsart University, was routinely cultured in tryptic soy broth (TSB; Thane (West), HiMedia, Maharashtra, India). The cultures were incubated in a shaking incubator at 30 °C and 160 rpm for 18 h. Following incubation, the bacterial cells were harvested via centrifugation at 6000× *g* for 10 min at 4 °C, washed twice with a sterile 0.85% NaCl solution, and resuspended to the desired concentration. The bacterial density was estimated by measuring the absorbance at an optical density (OD) of 600 nm (OD_600_) via a UV–Vis spectrophotometer (Thermo Fisher Scientific, Waltham, MA, USA). An absorbance of 1.0 corresponded to approximately 1.0 × 10^8^ CFU/mL according to a previously established laboratory calibration curve.

### 2.3. In Vitro Growth Assay of B. velezensis AAHM-BV2301 Under Different Treatments

The effects of different additives on the growth of *B. velezensis* AAHM-BV2301 were evaluated using an in vitro growth assay. Four treatments were prepared: Tryptic soy broth (TSB) supplemented separately with Delta-Ionic+™ (Ceresco Nutrition Inc., Saint-Régis, QC, Canada), silica nanoparticles (SiNPs) 95.74 ± 9.8 nm in size, chitosan (CS) (Krungthep Chemi, Bangkok, Thailand) and a combination of both additives (SiNPCS) at 2 mg/kg, 15 g/kg and 2 mg/kg + 15 g/kg. The treatment, which was not supplemented with any chemicals, served as the control. All treatments were prepared in triplicate (*n* = 3), thoroughly mixed, and sterilized by autoclaving at 121 °C for 15 min before inoculation. A standardized *B. velezensis* culture adjusted to an OD_600_ of 0.3, which is equivalent to approximately 3 × 10^7^ CFU/mL, was used as the inoculum. Exactly 1 mL of this standardized culture was introduced into 25 mL of each treatment medium (≈4% *v*/*v*). The cultures were incubated at 30 °C with constant shaking at 150 rpm for 48 h, and bacterial growth was monitored at 0, 12, 24, 36, and 48 h and estimated by measuring the absorbance at an OD_600_ as described above.

### 2.4. Experimental Diet Preparation

A commercial pellet diet (Uni-President, Di An, Binh Duong, Vietnam) containing 44% crude protein, 7% crude fat, 11% moisture, 17% ash, and 3% crude fiber was used as the basal feed across all treatment groups. Silica nanoparticles (SiNPs) and chitosan (CS), as mentioned above, were prepared for dietary inclusion at 2 mg/kg and 15 g/kg feed, respectively [18,19]. The functional diets were prepared by top-coating the basal feed with a *B. velezensis* suspension and the selected additives via a polyethylene bag mixing technique. Specifically, the feed was combined with *B. velezensis* at 1 × 10^8^ CFU/kg, along with SiNPs and/or chitosan, depending on the treatment group. The dietary treatments included the following: control (basal feed coated with 0.85% NaCl), BV (*B. velezensis* only), BVSiNP (BV + SiNPs), BVCS (BV + chitosan), and BVSiNPCS (BV + SiNPs + CS). After thorough manual mixing in sterile polyethylene bags to ensure uniform coating, the coated feed samples were homogenized in sterile 0.85% NaCl, serially diluted, and plated on TSA to confirm the targeted *B. velezensis* CFU/kg. The feeds were stored at 4 °C, and to maintain consistency and microbial viability, all the diets were freshly prepared daily throughout the experimental period.

### 2.5. Experimental Fish and Acclimation Procedures

Fingerling Asian seabass with an initial average weight of 7.25 ± 1.60 g were obtained from a commercial hatchery in Chachoengsao Province, Thailand. Upon arrival, the fish were subjected to a prophylactic bath in 100 ppm formalin for five minutes to minimize the risk of external infection [25]. The fish were subsequently acclimatized for two weeks in a 3000 L tank containing 20 ppt seawater with continuous aeration. During the acclimation period, the salinity was gradually reduced to 5 ppt. The fish were fed a commercial pellet diet twice daily, and their health status was assessed through routine behavioral observations and gross physical examinations.

### 2.6. Experimental Design and Feeding Trial

A completely randomized design (CRD) was used to evaluate the effects of dietary treatments on fingerling Asian seabass. Following acclimation, the fish whose average body weight was 11.25 ± 2.12 g were randomly assigned to five dietary groups: the control, BV, BVSiNP, BVCS, and BVSiNPCS groups. Each treatment group consisted of four replicates, with 20 fish per replicate. The fish were reared in 250 L high-density polyethylene (HDPE) tanks filled with 200 L of 5 ppt seawater and maintained under a static water system with continuous aeration. To ensure water quality, 20% of the water was exchanged daily.

Water quality parameters were monitored daily and maintained within optimal ranges: temperature, 28–30 °C; dissolved oxygen, >5 mg/L; pH, 7.5–8.0; and salinity, 5 ppt. The fish were fed their respective diets at 4% body weight per day and divided into three feeding sessions at 07:00, 12:00, and 17:00 for a total duration of 30 days. Uneaten feed and fecal matter were siphoned out daily, and a 12 h light:12 h dark photoperiod was maintained throughout the trial.

### 2.7. Sample Collection

#### 2.7.1. Sampling and Tissue Collection

At the end of the 30-day feeding trial, two fish per replicate (*n* = 8 per treatment) were randomly selected for sampling. The fish were sedated with clove oil (Hong Huat Co., Ltd., Bangkok, Thailand) at a concentration of 40 ppm [26]. Blood was collected via caudal vein puncture using sterile 1 mL syringes fitted with 23-G needles. The serum was separated via centrifugation at 8500 rpm for 15 min at 25 °C and stored at −80 °C for subsequent immunological and biochemical analyses.

Following blood collection, the fish were euthanized, and immune-related organs—including the liver, spleen, head kidney, gills, and intestine—were aseptically dissected. The tissue samples used for gene expression analysis were briefly preserved, and total RNA was extracted using easy-BLUE™ Total RNA Extraction Kits (iNtRON Biotechnology, Seongnam, Republic of Korea) following the manufacturer’s protocol. The samples intended for histological evaluation were fixed in 10% neutral buffered formalin (NBF).

#### 2.7.2. Isolation of Peripheral Blood Leukocytes (PBLs)

Peripheral blood lymphocytes were isolated following the method described by [27], with slight modifications. Briefly, 1 mL of EDTA-treated blood was diluted with 2 mL of PBS (pH 7.4) and layered over 3 mL of Histopaque^®^ (Sigma-Aldrich, St. Louis, MO, USA). After centrifugation at 200× *g* for 30 min at 25 °C, the leukocyte layer was collected, washed with PBS, and centrifuged at 400× *g* for 10 min. The final cell pellet was resuspended in 1 mL of PBS for downstream analysis.

### 2.8. Assessment of Growth Performance Parameters

The fish were weighed using an electronic digital scale to evaluate growth performance. The growth performance parameters were calculated via the following standard formulas to determine the effects of the experimental diets:

(1) Weight gain (WG, g) = Wt − Wi
(2) Average daily gain (ADG, g/fish/day) = (Wt − Wi)/t
(3) Relative growth rate (RGR, %) = [(Wt − Wi)/Wi] × 100
(4) Specific growth rate (SGR, %/day) = [ln(Wt) − ln(Wi)]/t × 100
(5) Feed conversion ratio (FCR) = total feed intake/WG

where Wt is the final body weight (g), Wi is the initial body weight (g), and t is the duration of the trial in days.

### 2.9. Immunological Assays

Three nonspecific immune parameters were evaluated in serum samples from previously collected fish. These included lysozyme activity, respiratory burst, and bactericidal activity, following the protocols of [9].

#### 2.9.1. Lysozyme Activity

Serum lysozyme activity was determined via a turbidimetric assay based on the lysis of *Micrococcus lysodeikticus*. Briefly, 10 μL of fish serum was added to 90 μL of *M. lysodeikticus* suspension (0.05 mg/mL in phosphate-buffered saline, pH 6.2) in 96-well microplates. The absorbance, OD, was measured at 540 nm immediately (0 min) and after 5 min of incubation at room temperature. Lysozyme activity was expressed as the change in optical density (ΔOD):

Lysozyme activity (ΔOD) = OD540 nm, 0 min − OD540 nm, 5 min

#### 2.9.2. Respiratory Burst Activity

Peripheral blood leukocytes (PBLs) from Section 2.7.2 were diluted, and 175 μL of the cell suspension was incubated with 25 μL of nitroblue tetrazolium (NBT) solution in the dark for 2 h at room temperature. After incubation, the cells were washed with 2 N KOH and methanol. The resulting supernatant was collected, and the absorbance was measured at 655 nm to quantify reactive oxygen species (ROS) production.

#### 2.9.3. Bactericidal Activity

Serum bactericidal activity from the previous section was assessed by incubating 40 μL of fish serum with *Vibrio vulnificus* (1 × 10^5^ CFU/mL) at 28 °C for 90 min. The mixture was then plated on thiosulfate citrate bile–salt sucrose (TCBS) agar, and the number of colony-forming units (CFUs) was calculated after 24 h. The bactericidal activity (%) was calculated via the following formula:

BA = [(T_0_ − T_24_)/T_0_] × 100,

where T_0_ and T_24_ represent the initial and final bacterial counts, respectively.

### 2.10. Quantification of Serum Biochemical Parameters

Serum biochemical parameters were quantified via an MSC100V veterinary chemistry analyzer (PushKang Biotechnology, Hangzhou, Zhejiang, China) [28]. A total of 23 parameters, including albumin (ALB), total protein (TP), alkaline phosphatase (ALP), alanine aminotransferase (ALT), aspartate aminotransferase (AST), gamma-glutamyl transferase (GGT), direct bilirubin (DBIL), total bilirubin (TBIL), globulin (GLB), indirect bilirubin (IBIL), the albumin/globulin ratio (ALB/GLB), calcium (Ca), creatinine (Crea), total carbon dioxide (tCO_2_), phosphorus (P), cholesterol (CHOL), glucose (GLU), urea (Ure), amylase (AMY), creatine kinase (CK), the urea-to-creatinine ratio (Urea/Crea), total bile acids (TBA), and the AST-to-ALT ratio (AST/ALT), were assessed via manufacturer-supplied reagent kits.

### 2.11. Total RNA Extraction, cDNA Synthesis, and Quantitative Real-Time PCR for Immune Gene Expression

Total RNA was extracted from approximately 20 mg of gill, spleen, intestine, liver, and head kidney tissues or 100 μL of PBLs from the previous section via easy-BLUE™ Total RNA Extraction Kits according to the manufacturer’s instructions. The RNA concentration and purity were assessed via a NanoDrop™ 2000 spectrophotometer (Thermo Scientific, Waltham, MA, USA) and adjusted to 50 ng/μL. First-strand cDNA synthesis was conducted using Maxime™ RT PreMix (iNtRON Biotechnology, Seongnam, Republic of Korea) with 20 μL of total RNA. Reverse transcription was performed at 45 °C for 60 min, followed by enzyme inactivation at 95 °C for 5 min. The resulting cDNA was diluted 1:1 with DEPC-treated water for downstream use.

Quantitative real-time PCR (qRT–PCR) was carried out using an AriaMx Real-Time PCR system (Agilent Technologies, Santa Clara, CA, USA). The first-strand cDNA of the above section was carefully subjected to an identical reaction. Each 10 μL reaction contained 2 μL of cDNA, 1 μL each of forward and reverse primers, 5 μL of RealMOD™ Green W^2^ 2× qPCR mix (iNtRON Biotechnology, Gyeonggi-do, Republic of Korea), and 1 μL of DEPC-treated water. The cycling protocol was as follows: 95 °C for 5 min, followed by 30 cycles of 95 °C for 30 s, 60 °C for 30 s, and 72 °C for 30 s, with a final extension at 72 °C for 30 s. The target genes included *Hep-1*, *α2M*, *C3*, *CC*, *Lyz*, *HSP70*, *IL-8*, and *Mx*, with *β-actin* and *18S rRNA* as internal reference genes. The primer sequences are provided in Supplementary Material Table S1. Relative gene expression levels were calculated via the 2^−ΔΔCt^ method [29].

### 2.12. Gut Microbiota Profiling and Bioinformatics Analysis

#### 2.12.1. High-Throughput 16S Ribosomal RNA Gene Sequencing

At the end of the feeding trials, the genomic DNA of the middle sections of the intestines was extracted using TIANamp DNA Kits (Tiangen Biotech, Beijing, China) according to the manufacturer’s instructions. The V1–V9 hypervariable regions of the 16S rRNA gene were amplified via the primers 27F (AGRGTTTGATYNTGGCTCAG) and 1492R (TASGGHTACCTTGTTASGACTT). Amplicons were quantified, normalized to equimolar concentrations, pooled, and sequenced on the PacBio Sequel II platform (Beijing Biomarker Technologies Co., Ltd., Beijing, China).

#### 2.12.2. Bioinformatics Analysis of Microbiome Composition and Diversity

High-quality sequences exhibiting ≥97% similarity were clustered into operational taxonomic units (OTUs) via USEARCH (v10.0). The taxonomic classification of OTUs was conducted in QIIME2 [30] by employing a naive Bayes classifier trained on the SILVA reference database (release 138.1) [31] with a minimum confidence threshold of 70%. Alpha diversity metrics were calculated to assess within-sample species richness and evenness, whereas beta diversity was evaluated via principal coordinate analysis (PCoA) to determine compositional dissimilarity among samples. One-way analysis of variance (ANOVA) was used to compare bacterial abundance and diversity across treatments. Linear discriminant analysis effect size (LEfSe) was applied to identify taxa with significantly differential abundance [32]. All bioinformatics and statistical analyses were conducted via the BMKCloud platform (https://www.biocloud.net).

### 2.13. Histological Analysis and Intestinal Morphometry

Liver and intestine samples were fixed in 10% neutral buffered formalin, embedded in paraffin, and sectioned at 3–5 μm using a rotary microtome. The sections were stained with hematoxylin and eosin (H&E) following standard protocols [33], including deparaffinization in xylene, rehydration through graded ethanol, nuclear staining with Harris hematoxylin, differentiation with 1% acid alcohol, bluing in ammonia water, and cytoplasmic counterstaining with eosin. The stained slides were dehydrated, cleared, and mounted for examination under a light microscope (Olympus CX41, Tokyo, Japan).

Photomicrographs of intestinal sections were captured using a light microscope (Olympus CX41, Tokyo, Japan). Villus height was measured from the tip to the crypt junction in ten well-oriented villi per fish, and the goblet cells were manually enumerated in three villi per fish. Measurements were performed using ImageJ version 1.54 g (NIH, Bethesda, MD, USA), and goblet cell counts are expressed as the number of cells per villus.

### 2.14. Bacterial Challenge Test

At the end of the feeding trial, disease resistance against *Vibrio vulnificus* was evaluated following the procedure of [34] with minor modifications. The *V. vulnificus* strain AAHM-VV2410 used in this study was acquired from the culture collection of the CE AAHM. Briefly, the bacterium was cultured in tryptic soy broth (TSB) supplemented with 2% NaCl at 37 °C for 18 h, harvested, washed, and resuspended in sterile 0.85% NaCl. The suspension was adjusted to 1 × 10^6^ CFU/mL by the methods described by [31].

Five experimental groups were used, with fifteen fish per group randomly selected from the feeding trial. Each fish was intraperitoneally injected with 100 μL of the bacterial suspension (equivalent to 1 × 10^5^ CFU/fish) and then transferred to 250 L HDPE tanks containing 200 L of 5 ppt water. The fish were monitored regularly, and mortality was recorded daily for 14 days. Dead fish were promptly removed to prevent deterioration of water quality.

### 2.15. Statistical Analysis

All the statistical analyses were conducted using SPSS for Mac (version 24.0; IBM Corp., Armonk, NY, USA). Data were first assessed for normality and homogeneity of variance via the Shapiro–Wilk test and Levene’s test, respectively. One-way analysis of variance (ANOVA) followed by Tukey’s post hoc test was employed to evaluate significant differences among treatment groups. The results are expressed as the means ± standard deviations (SDs), with statistical significance accepted at *p* < 0.05. Significant group differences were visualized via either compact letter display (CLD) or asterisk annotations (* *p* < 0.05, ** *p* < 0.01, *** *p* < 0.001, **** *p* < 0.0001), depending on the figure presentation. Postchallenge survival data were analyzed via the Kaplan–Meier method, and group comparisons were performed via the log-rank (Mantel–Cox) test.

## 3. Results

### 3.1. In Vitro Growth of Bacillus velezensis AAHM-BV2301 Under Various Conditions

**Figure 1:**
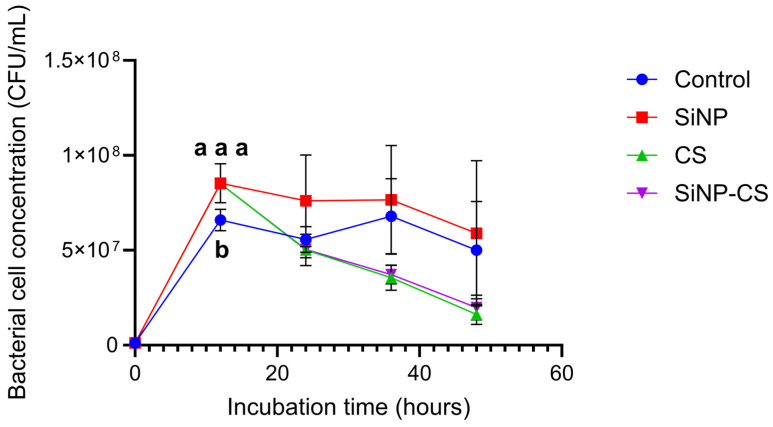
In vitro growth of *Bacillus velezensis* AAHM-BV2301 under different treatments of silica nanoparticles (SiNPs), chitosan (CS), and their combination (SiNP–CS) compared with the control. Bacterial growth was determined as the viable cell count (CFU/mL) after 12, 24, 36, and 48 h of incubation at 30 °C using a UV–Vis spectrophotometer. The values are the means ± SDs (*n* = 3). Different letters at h 12 of the incubation period indicate a significant difference (*p* < 0.05).

The growth of *B. velezensis* AAHM-BV2301 differed among the treatments (Figure 1). All the cultures started at approximately 1.18 × 10^6^ ± 2.08 × 10^4^ CFU/mL at 0 h and markedly increased after 12 h of incubation. At this time, the SiNP group had the highest viable count (8.54 × 10^7^ ± 1.03 × 10^7^ CFU/mL), followed by the CS (8.51 × 10^7^ ± 1.08 × 10^7^ CFU/mL), SiNPCS (8.51 × 10^7^ ± 1.08 × 10^6^ CFU/mL), and control (6.59 × 10^7^ ± 5.65 × 10^6^ CFU/mL) groups. Compared with the control treatment, all supplemented treatments resulted in significantly greater viable counts at 12 h (*p* < 0.05). After 24 h, growth plateaued in all the treatments but gradually decreased until 48 h.

### 3.2. Growth Performance

**Figure 2:**
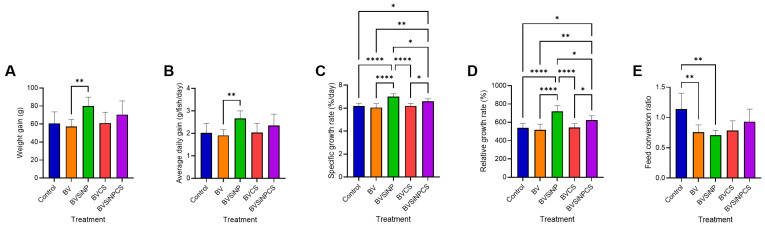
Growth performance metrics of Asian seabass fed probiotic and synbiotic diets. (**A**) Weight gain (g), (**B**) average daily gain (g/fish/day), (**C**) specific growth rate (SGR, %/day), (**D**) relative growth rate (RGR, %), and (**E**) feed conversion ratio (FCR) across five dietary treatments: control (basal diet), *Bacillus velezensis* (BV), BV + silica nanoparticles (BVSiNP), BV + chitosan (BVCS), and BV + silica nanoparticles + chitosan (BVSiNPCS). The values are expressed as the means ± standard deviations. Significant differences are denoted by asterisks (* *p* < 0.05, ** *p* < 0.01, **** *p* < 0.0001).

Dietary treatments significantly influenced the growth performance of Asian seabass (Figure 2). The weight gain (60.75 ± 12.71 g; Figure 2A) and average daily gain (2.66 ± 0.33 g/fish/day; Figure 2B) were significantly greater in the fish fed the BVSiNP diet than in those fed the BV diet (*p* < 0.01). The specific growth rate (7.00 ± 0.26%/day; Figure 2C) and relative growth rate (718.50 ± 62.50%; Figure 2D) were also significantly greater than those of the control, BV, and BVCS groups (*p* < 0.0001), whereas the BVSiNPCS group presented intermediate but significantly improved values (*p* < 0.05–0.01). The feed conversion ratio was lowest in the BVSiNP group (0.71 ± 0.08; Figure 2E), followed by that in the BV group (0.76 ± 0.12), both of which were significantly lower than that in the control group (1.14 ± 0.26; *p* < 0.01). The survival rate of all treatments was 100% with no mortality during the experimental periods.

### 3.3. Serum Biochemical Responses

**Figure 3:**
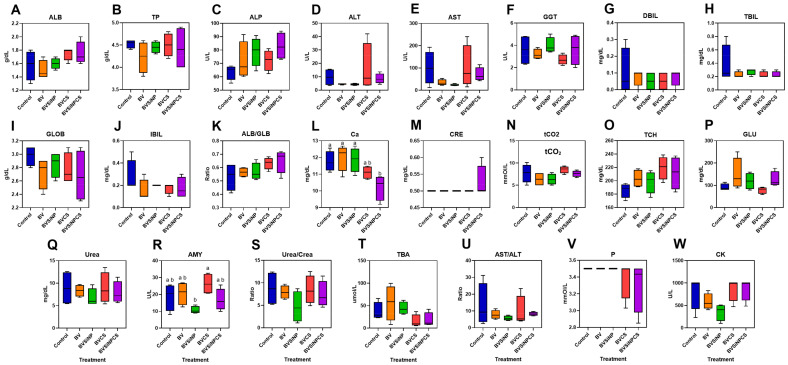
Serum biochemical parameters of Asian seabass, namely, albumin (ALB) (**A**), total protein (TP) (**B**), alkaline phosphatase (ALP) (**C**), alanine aminotransferase (ALT) (**D**), aspartate aminotransferase (AST) (**E**), gamma-glutamyl transferase (GGT) (**F**), direct bilirubin (DBIL) (**G**), total bilirubin (TBIL) (**H**), globulin (GLOB) (**I**), indirect bilirubin (IBIL) (**J**), the albumin/globulin ratio (ALB/GLB) (**K**), calcium (Ca) (**L**), creatinine (CRE) (**M**), total carbon dioxide (tCO_2_) (**N**), total cholesterol (TCH) (**O**), glucose (GLU) (**P**), urea (Urea) (**Q**), amylase (AMY) (**R**), the urea-to-creatinine ratio (Urea/Crea) (**S**), total bile acids (TBA) (**T**), the AST-to-ALT ratio (AST/ALT) (**U**), phosphorus (P) (**V**), creatine kinase (CK) (**W**), and, were evaluated across five dietary treatments: control (basal diet), Bacillus velezensis (BV), BV + silica nanoparticles (BVSiNP), BV + chitosan (BVCS), and BV + silica nanoparticles + chitosan (BVSiNPCS). The data are presented as box-and-whisker plots (*n* = 4). The lowercased letter display (CLD) annotations indicate statistically significant group differences (*p* < 0.05).

Among the 23 biochemical parameters analyzed (Figure 3A–W), 21 were not significantly different among the treatment groups, indicating minimal systemic alterations; however, calcium levels were significantly lower in the BVSiNPCS group than in the control, BV, and BVSiNP groups (*p* < 0.05; Figure 3L), whereas amylase activity was significantly lower in the BVSiNP group and highest in the BVCS group (*p* < 0.05; Figure 3R).

### 3.4. Nonspecific Immune Responses

**Figure 4:**
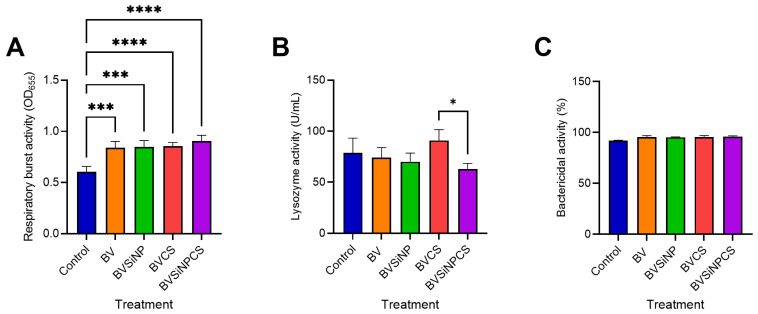
Nonspecific innate immune responses in the serum of Asian seabass fed *B. velezensis*-based functional diets. (**A**) Respiratory burst activity (OD_655_), (**B**) lysozyme activity (U/mL), and (**C**) bactericidal activity (%) against *Vibrio vulnificus*. Dietary treatments: Control (basal diet), BV (*B. velezensis*), BVSiNP (BV + SiNPs), BVCS (BV + Chitosan), and BVSiNPCS (BV + SiNPs + Chitosan). The values represent the means ± SDs (*n* = 4). Asterisks denote significant differences compared with the control group (* *p* < 0.05, *** *p* < 0.001, **** *p* < 0.0001).

Dietary treatments significantly influenced nonspecific immune responses in Asian seabass (Figure 4). Compared with that in the control group, respiratory burst activity was markedly elevated in all the treated groups (Figure 4A), with highly significant increases in BV and BVSiNP (*p* < 0.001), and BVCS and BVSiNPCS exhibited even greater responses (*p* < 0.0001). Lysozyme activity (Figure 4B) was highest in the BVCS group and significantly exceeded that in the BVSiNPCS group (*p* < 0.05). In contrast, the bactericidal activity remained consistently high (91.95–96.09%) across all the groups, with no significant differences observed (Figure 4C).

### 3.5. Immune-Related Gene Expression

**Figure 5:**
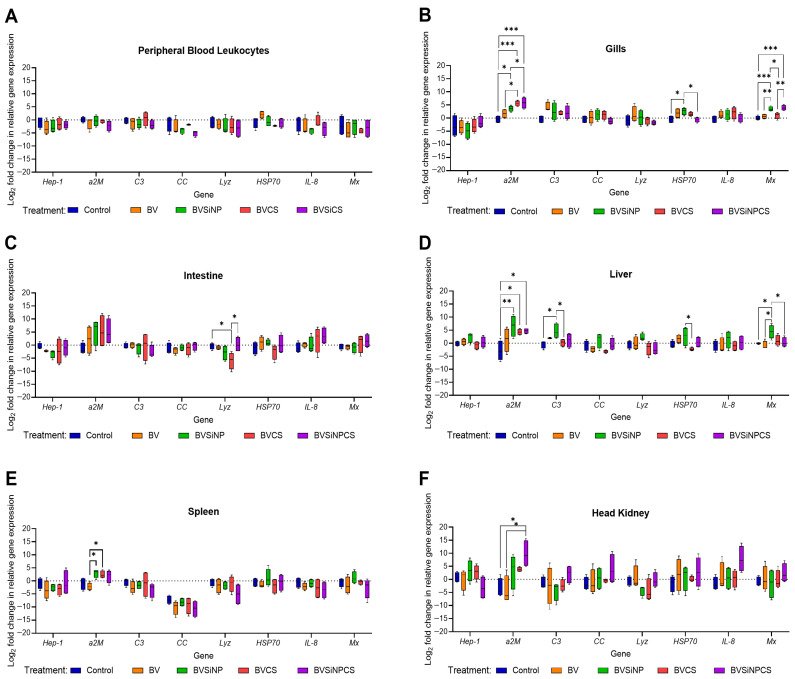
Expression analysis of immune-related genes (log_2_-fold change vs. control) in (**A**) peripheral blood leukocytes, (**B**) gills, (**C**) intestine, (**D**) liver, (**E**) spleen, and (**F**) head kidney of Asian seabass across five treatments: control (basal diet), *Bacillus velezensis* (BV), BV + silica nanoparticles (BVSiNP), BV + chitosan (BVCS), and BV + silica nanoparticles + chitosan (BVSiNPCS) (*n* = 4). The target genes included hepcidin-1 (*Hep-1*), alpha-2-macroglobulin (*a2M*), complement component 3 (*C3*), CC-chemokine (*CC*), lysozyme (*Lyz*), heat shock protein 70 (*HSP70*), interleukin-8 (*IL-8*), and myxovirus resistance protein (*Mx*). Asterisks denote significant differences (* *p* < 0.05, ** *p* < 0.01, *** *p* < 0.001).

Immune gene expression varied across tissues in response to dietary treatments (Figure 5A–F). No significant differences were detected in peripheral blood leukocytes (Figure 5A), indicating limited systemic modulation. In contrast, the response of gill tissue (Figure 5B) was strong, particularly in the BVSiNP, BVCS, and BVSiNPCS groups. *a2M* expression was significantly greater in the BVSiNP group (*p* < 0.05) and highly significantly greater in the BVCS and BVSiNPCS groups than in the control group (*p* < 0.001). *HSP70* expression was significantly upregulated in the BVSiNP group compared with that in the control and BVSiNPCS groups (*p* < 0.05). *Mx* expression was significantly greater in the BVSiNP and BVSiNPCS groups than in the control group (*p* < 0.001), with additional significant differences among the BV, BVCS, and BVSiNP groups (*p* < 0.01–0.05).

In the intestine (Figure 5C), *a2M*, *IL-8*, and *Mx* were seemingly upregulated in the BVCS and BVSiNPCS groups, although the differences were not statistically significant. However, *lyz* was significantly lower in the BVCS group than in both the control and the BVSiNP groups (*p* < 0.05). In the liver (Figure 5D), *a2M* was significantly elevated in the BVSiNP group (*p* < 0.01), as well as in the BVCS and BVSiNPCS groups (*p* < 0.05). *C3* and *HSP70* were significantly upregulated in the BVSiNP group (*p* < 0.05), whereas *Mx* was broadly upregulated across the BVSiNP, BVSiNPCS, and control groups (*p* < 0.05).

In the spleen (Figure 5E), *a2M* expression was significantly greater in the BVSiNP and BVCS groups than in the BV group (*p* < 0.05). In the head kidney (Figure 5F), *a2M* was significantly upregulated in the BVSiNPCS group compared with the control and BV groups (*p* < 0.05), indicating tissue-specific immune modulation by the synbiotic formulations.

### 3.6. Investigation of the Gut Microbiota

High-throughput sequencing of the *16S rRNA* gene generated a total of 713,965 raw circular consensus sequences (CCSs) across all the samples. After quality filtering, primer trimming, and chimera removal, 711,098 high-quality sequences remained, with an average of 35,555 ± 2798 sequences per sample. The mean sequence length was 1448 ± 14 bp, covering nearly the full length of the *16S rRNA* gene (Supplementary Material Table S2). The rarefaction curves for all the samples reached a plateau, indicating sufficient sequencing depth to capture the majority of the microbial diversity (Supplementary Materials Figure S1). The sequences were clustered into 1218 operational taxonomic units (OTUs) at a 97% sequence identity threshold, with an average of 162 ± 203 OTUs per sample.

### 3.7. Taxonomic Composition

**Figure 6:**
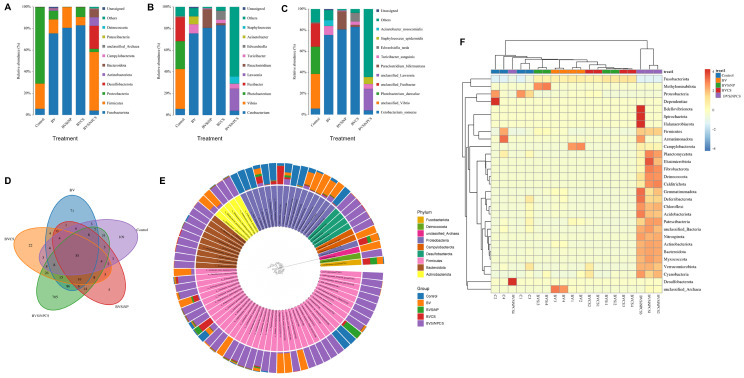
Composition and distribution of the intestinal microbiota in Asian seabass (*n* = 4). Relative abundance at the (**A**) phylum, (**B**) genus, and (**C**) species levels on the basis of *16S rRNA* gene sequencing. (**D**) Venn diagram of shared and unique operational taxonomic units (OTUs). (**E**) Circular cladogram showing phylogenetic relationships and hierarchical taxonomic distributions across treatments. (**F**) Heatmap of the 35 most abundant taxa, with color intensity representing relative abundance. Dietary treatments: Control (basal diet), BV (*B. velezensis*), BVSiNP (BV + SiNPs), BVCS (BV + Chitosan), and BVSiNPCS (BV + SiNPs + Chitosan).

At the phylum level (Figure 6A), Fusobacteriota, Firmicutes, Proteobacteria, and Desulfobacterota dominated the gut microbiota across all groups, accounting for more than 95.6% of the total sequences. The control group was enriched in Proteobacteria (70.6%), whereas the BV, BVSiNP, and BVCS groups were predominantly enriched in Fusobacteriota (75.4–82.9%), followed by Firmicutes and Proteobacteria. In contrast, BVSiNPCS presented a distinct profile, with Firmicutes (54.2%), Desulfobacterota (21.3%), and Actinobacteriota (7.9%) dominating and a marked reduction in Fusobacteriota (4.0%).

At the genus level (Figure 6B), *Vibrio*, *Photobacterium*, and *Fusibacter* were prevalent in the control group, whereas *Cetobacterium* dominated the BV, BVSiNP, and BVCS groups (75.4–82.9%). BVSiNPCS exhibited a divergent composition, with *Lawsonia* (20.3%) and *Staphylococcus* (6.6%) present among a large proportion of unclassified taxa (64.3%).

Species-level analysis further revealed that *Cetobacterium somerae* was highly abundant in the BV, BVSiNP, and BVCS groups (>75%), whereas the control group contained unclassified *Vibrio*, *Photobacterium damselae* and unclassified *Fusibacter* (Figure 6C). The BVSiNPCS group was primarily composed of unclassified taxa (64.4%), *Lawsonia* spp. (20.3%), and *Staphylococcus epidermidis* (6.6%), indicating a unique microbial profile.

A Venn diagram of the OTUs (Figure 6D) revealed 35 core taxa shared among all the groups, with BVSiNPCS harboring the most unique OTUs (705), followed by the control (109) and BV (71) groups. The BVSiNP group had the fewest unique taxa (5) but overlapped substantially with the BV and BVSiNPCS groups. Overlaps between BVCS and BVSiNPCS indicate that a converging microbiota is influenced by synbiotic supplementation. The phylogenetic distribution and relative abundance of the dominant gut microbiota across treatments are further visualized in a circular cladogram (Figure 5E), which illustrates treatment-specific taxonomic divergence. Notably, the BVSiNPCS group presented a distinct microbial composition characterized by a predominance of Firmicutes, Bacteroidota, and Desulfobacterota, whereas the control group was enriched with Proteobacteria. In contrast, the Fusobacteriota-dominated profiles, specifically those of *Cetobacterium somerae*, were observed in the BV, BVSiNP, and BVCS groups.

Phylum-level heatmap analysis revealed clear clustering patterns (Figure 6F). The BVSiNPCS group presented the greatest microbial diversity and was enriched in rare phyla, such as Bdellovibrionota, Spirochaetota, and Planctomycetota, whereas the control and BV groups presented lower microbial diversity. Synbiotic treatments, particularly BVCS and BVSiNPCS, clustered together, reflecting similar shifts in the gut microbial composition.

### 3.8. Alpha Diversity of the Gut Microbiota

**Figure 7:**
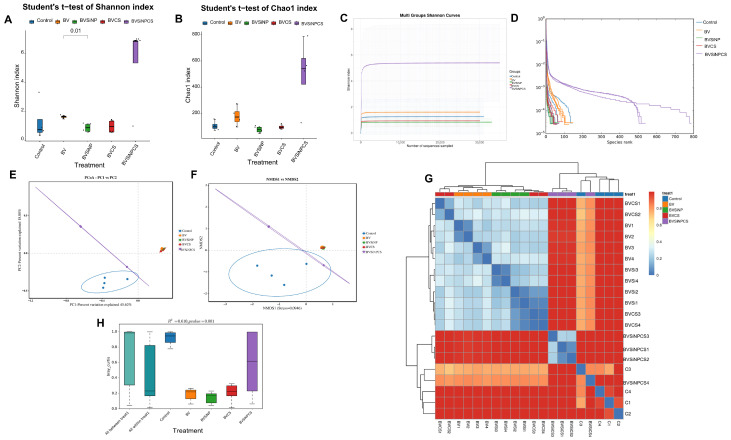
Alpha and beta diversity metrics of the intestinal microbiota in Asian seabass. (**A**) Shannon index and (**B**) Chao1 index comparing alpha diversity among groups. (**C**) Shannon and (**D**) rank abundance curves. (**E**) Principal component analysis (PCA) and (**F**) nonmetric multidimensional scaling (NMDS) plots based on Bray–Curtis dissimilarity. (**G**) Heatmap of Bray–Curtis pairwise dissimilarities with hierarchical clustering. (**H**) Boxplot of within-group Bray–Curtis distances indicating microbial community dispersion. Dietary treatments: Control (basal diet), BV (*B. velezensis*), BVSiNP (BV + SiNPs), BVCS (BV + Chitosan), and BVSiNPCS (BV + SiNPs + Chitosan).

Alpha diversity metrics revealed differences in microbial diversity among the treatment groups (Figure 7A,B). Compared with the other groups, the BVSiNPCS group presented the greatest diversity, with elevated Shannon (5.39 ± 2.97) and Chao1 (499.54 ± 275.63) indices (Figure 7A,B), indicating greater species richness and evenness. In contrast, compared with the BVSiNP group (0.84 ± 0.30; *p* < 0.01), the BV group presented significantly greater Shannon diversity (1.61 ± 0.11), indicating a reduction in microbial evenness with the addition of silica nanoparticles alone. Overall, the control, BV, and BVSiNP groups presented lower diversity values.

Shannon rarefaction curves (Figure 7C) confirmed that the BVSiNPCS group presented the highest microbial diversity, with curves plateauing above 5, indicating a complex and balanced gut community. Similarly, a rank-abundance analysis (Figure 7D) revealed greater richness and evenness in the BVSiNPCS group than in the other treatment groups, further supporting its enhanced microbial diversity and ecological stability.

### 3.9. Beta Diversity of the Gut Microbiota

Beta diversity analyses revealed significant differences in the gut microbial composition among the treatment groups. Both the PCoA and NMDS plots (Figure 7E,F) displayed distinct clustering, with the control group clearly separated from the synbiotic and nanoparticle-supplemented groups. The Bray–Curtis dissimilarity heatmap (Figure 7G) revealed high intragroup similarity and strong intergroup separation, particularly between the BVSiNPCS and control groups. These observations were supported by PERMANOVA (Figure 7H), which confirmed a significant effect of dietary treatment on the microbial community structure (R^2^ = 0.610, *p* = 0.001). Notably, the BVSiNPCS group presented the greatest within-group variability, reflecting complex and dynamic microbial shifts associated with combined synbiotic and nanoparticle supplementation.

### 3.10. Differentially Abundant Microbial Taxa

**Figure 8:**
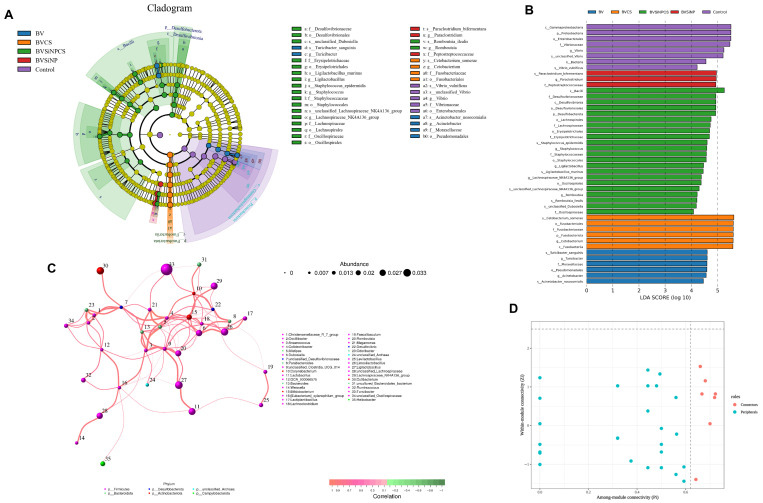
Differential abundance and microbial co-occurrence network analysis of the gut microbiota in Asian seabass. (**A**) LEfSe-derived cladogram highlighting taxonomic biomarkers for each dietary group: control (purple), BV (blue), BVCS (orange), BVSiNPCS (red), and BVSiNP (green). Node size represents relative abundance; color indicates group-specific enrichment. (**B**) LDA (log_10_) scores of significantly enriched taxa (LDA > 4.0). (**C**) Correlation network of genera based on Spearman correlations (r > 0.1, *p* < 0.05); node size = abundance, edge thickness = correlation strength, edge color = correlation direction (red = positive, green = negative), node color = phylum. (**D**) Zi–Pi plot indicating topological roles: peripherals (blue) and connectors (pink), with classification thresholds of Zi = 2.5 and Pi = 0.62.

LEfSe revealed distinct microbial biomarkers associated with each dietary treatment in Asian seabass, with all the taxa surpassing the LDA score threshold (log_10_ > 2), indicating treatment-specific modulation of the gut microbiota (Figure 8A,B). The BVSiNPCS group presented the greatest number of discriminative taxa, including the *Lachnospiraceae NK4A136* group, *Staphylococcus epidermidis*, *Romboutsia ilealis*, and members of *Bacilli* and *Desulfobacteriota*. The BVCS group was dominated by *Fusobacteriota*, particularly *Cetobacterium somerae*. The BVSiNP group was enriched in *Paraclostridium bifermentans* and *Peptostreptococcaceae*, whereas the BV group was enriched in *Turicibacter sanguinis* and *Acinetobacter*. In contrast, the control group was enriched in *Proteobacteria*, notably *Vibrionaceae* and *Vibrio vulnificus*.

### 3.11. Microbial Co-Occurrence Network Analysis

The microbial co-occurrence network (Figure 8C) constructed from the gut microbiota profiles revealed a highly modular and positively correlated structure comprising multiple strong associations among bacterial taxa. A total of 12 nodes and 15 edges were identified, with all the edges representing statistically significant positive correlations (Spearman’s ρ ≥ 0.89, *p* < 1.9 × 10^−7^). Notably, a triadic cluster involving *Christensenellaceae*_R_7_group, *Oscillibacter*, and *Odoribacter* exhibited perfect pairwise correlations (ρ = 1.000, *p* < 0.001), with a maximum edge weight of 6. This triad formed the central core of the network, indicating tight ecological linkages.

Other high-strength correlations were observed between *Anaerococcus* and *Alistipes* (ρ = 0.932; *p* = 2.29 × 10^−9^), between *Colidexribacter* and *Megamonas* (ρ = 0.902; *p* = 5.75 × 10^−8^), and between *Dubosiella* and *Limosilactobacillus* (ρ = 0.895; *p* = 1.02 × 10^−7^). These interactions were supported by substantial edge weights (5.6–5.9), indicating robust coabundance relationships.

Node role classification on the basis of within-module connectivity (Zi) and among-module connectivity (Pi) (Figure 8D) revealed that most taxa, including Christensenellaceae_R_7_group, *Oscillibacter*, and *Cutibacterium*, functioned as peripheral nodes (Zi ≤ 2.5, Pi ≤ 0.62), indicating limited intermodular connectivity. In contrast, unclassified_Desulfovibrionaceae, *Odoribacter*, and *Anaerococcus* were identified as nonhub connectors (Zi ≤ 2.5, Pi > 0.62), resulting in a bridging role between distinct microbial modules. The greatest node size and connectivity were observed in unclassified_Clostridia_UCG_014 and *Colidextribacter*, supporting their central roles within the network structure. These findings demonstrate that dietary modulation resulted in a structured and cooperative microbial network, with Firmicutes and Bacteroidota members forming dominant clusters and exhibiting strong intertaxa connectivity.

### 3.12. Functional Annotation of Microbial Communities

**Figure 9:**
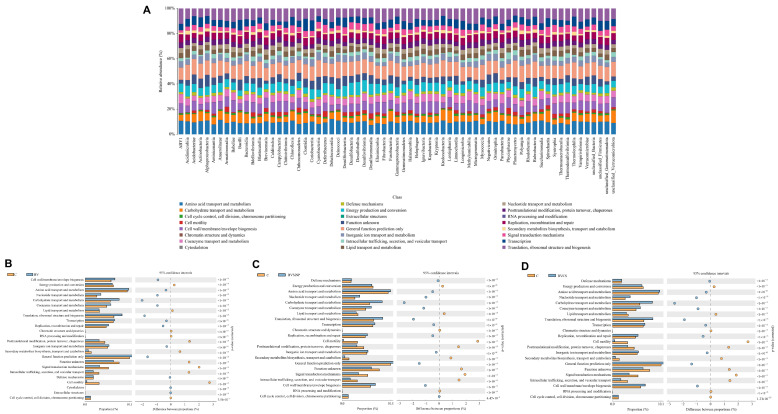
Functional prediction of intestinal microbiota in Asian seabass based on Clusters of Orthologous Groups (COG) classification. (**A**) Stacked bar plots of predicted COG functions across treatments. (**B**) Control vs. BV (*B. velezensis*), (**C**) control vs. BVSiNP (BV + SiNPs), and (**D**) control vs. BVCS (BV + chitosan) comparisons showing functional shifts with 95% confidence intervals.

COG-based functional annotation revealed broad metabolic and cellular roles across microbial taxa, with dominant functions such as amino acid transport and metabolism, carbohydrate transport and metabolism, energy production, cell wall/membrane/envelope biogenesis, transcription, and translation (Figure 9A). Significant shifts in functional profiles were observed between the control and BV-based treatment groups (Figure 9B–D). All the BV-supplemented groups (BV, BVCS, and BVSINP) presented consistent and significant enrichment (*p* < 1.0 × 10^−15^) in key functional categories: cell wall/membrane/envelope biogenesis; amino acid, nucleotide, and carbohydrate transport and metabolism; coenzyme transport and metabolism; translation, ribosomal structure and biogenesis; transcription; replication, recombination and repair; inorganic ion transport and metabolism; general function prediction only; and defense mechanisms. The BVSiNPCS group was excluded from the analysis because of high species richness and a large proportion of unclassified taxa, which impeded accurate COG annotation. These results indicate that BV-based probiotic and synbiotic supplementation substantially increased the biosynthetic, metabolic, and defense functional potential of the gut microbiome in Asian seabass.

### 3.13. Histological Assessment and Intestinal Morphometric Analysis

**Figure 10:**
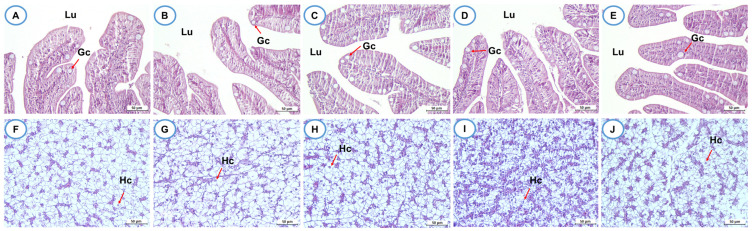
Histological examination of intestinal (**A**–**E**) and hepatic (**F**–**J**) tissues of Asian seabass following 30 days of dietary supplementation. (**A**–**E**) show cross-sections of the mid-intestine stained with hematoxylin and eosin (H&E), highlighting the intestinal lumen (Lu) and goblet cells (Gc). Panels (**F**–**J**) show liver sections, indicating hepatocyte nuclei (Hc) and normal hepatic architecture. The data correspond to the (**A**,**F**) control, (**B**,**G**) BV (*B. velezensis*), (**C**,**H**) BVSiNP (BV + SiNPs), (**D**,**I**) BVCS (BV + Chitosan), and (**E**,**J**) BVSiNPCS (BV + SiNPs + Chitosan) groups. All scale bars = 50 µm.

Histological examination of intestinal and liver tissues (Figure 10A–J) revealed no major structural alterations across all treatment groups. There was no evidence of inflammation, necrosis, or hemorrhage, indicating that the dietary treatments were nontoxic and well tolerated.

**Figure 11:**
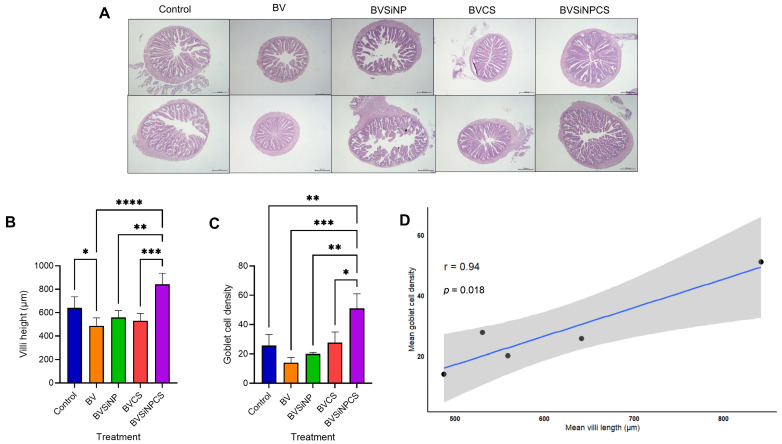
Histological and morphometric parameters of the intestine of Asian seabass following 30 days of probiotic and synbiotic supplementation. Dietary treatments: Control (basal diet), BV (*B. velezensis*), BVSiNP (BV + SiNPs), BVCS (BV + Chitosan), and BVSiNPCS (BV + SiNPs + Chitosan). (**A**) Histological samples of the intestine in each group. (**B**) Villus height (µm) and (**C**) goblet cell density (cells/villus) are presented as the mean ± SD. (**D**) Pearson’s correlation between mean villus height and goblet cell density across treatments. Asterisks indicate statistically significant differences among groups (* *p* < 0.05, ** *p* < 0.01, *** *p* < 0.001, **** *p* < 0.0001).

Histological samples of the intestine from each group are shown in Figure 11A. Significant differences in intestinal villus height and goblet cell density were observed among the dietary groups (Figure 11B,C). In the BVSiNPCS group, the villus height reached 842.27 ± 94.54 µm, which was a highly significant increase compared with that in all the other treatments (*p* < 0.01–0.001), whereas compared with the control group, the BV group presented a significantly lower villus height (*p* < 0.05) (Figure 11B). Goblet cell density was likewise markedly elevated in the BVSiNPCS group (51.00 ± 10.00 cells/villus), with significant differences relative to that in both the control group and all the other treatment groups (*p* < 0.05–0.001) (Figure 11C). Furthermore, a strong positive correlation was detected between villus height and goblet cell density (*r* = 0.94, *p* = 0.018) (Figure 11D).

### 3.14. Disease Resistance Against Vibrio Vulnificus

**Figure 12:**
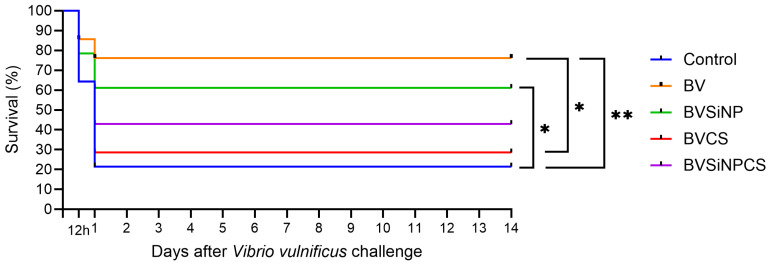
Kaplan–Meier survival curves of Asian seabass after 14 days of *V. vulnificus* challenge. Significant differences among dietary groups were determined by the log-rank test (* *p* < 0.05, ** *p* < 0.01). Dietary treatments: Control (basal diet), BV (*B. velezensis*), BVSiNP (BV + SiNPs), BVCS (BV + Chitosan), and BVSiNPCS (BV + SiNPs + Chitosan).

The postchallenge survival rates are shown in Figure 12. Compared with both the control (*p* < 0.01) and the BVCS (*p* < 0.05) groups, the survival of the BV-treated group was significantly greater. Compared with the control group, the BVSiNP group also had significantly improved survival (*p* < 0.05). In contrast, the BVSiNPCS and BVCS groups had moderate to low survival. Mortality occurred within 12–24 h, particularly in the control and BVCS groups, indicating high pathogen virulence. These findings indicate that *B. velezensis*, alone or in combination with silica nanoparticles, enhances disease resistance in Asian seabass against *V. vulnificus*.

## 4. Discussion

In vitro growth assays revealed that supplementation with SiNPs, CS, or their combination (SiNPCS) influenced the proliferation of *B. velezensis* AAHM-BV2301. Compared with the control treatment, all supplemented treatments resulted in significantly greater viable counts at 12 h (*p* < 0.05), suggesting that these additives stimulated early bacterial growth. In vitro assays revealed that CS produced an early, transient increase in *B. velezensis* but did not sustain growth beyond 24 h. This biphasic pattern suggests a concentration- and exposure-dependent effect in which CS can initially support proliferation yet exert mild inhibitory pressure with prolonged contact [16,17,19]. In contrast, SiNPs most effectively enhanced the exponential phase, plausibly by improving nutrient bioavailability and providing favorable attachment surfaces within this physicochemical environment [18,20].

Taken together, the responses indicate a potential synergistic interaction between SiNPs and *B. velezensis* under the present in vitro conditions.

An in vivo study demonstrated that dietary supplementation with *B. velezensis*, SiNPs, and chitosan, either individually or in combination, enhances growth performance, innate immunity, the gut microbiota composition, and disease resistance in Asian seabass. Among all the treatments, the BVSiNP formulation yielded the most pronounced improvements, indicating a synergistic effect between the probiotic and nanoparticles under in vivo conditions.

The exceptional growth performance in the BVSiNP group, characterized by increased WG, ADG, SGR, and RGR and reduced FCR, indicates functional synergy between *B. velezensis* and SiNPs. This increase likely results from complementary mechanisms: *B. velezensis* promotes digestion through the secretion of extracellular enzymes, such as proteases, lipases, and amylases, facilitating nutrient breakdown and assimilation [35,36,37]. Moreover, SiNPs improve nutrient bioavailability through their high surface-area-to-volume ratio and their ability to activate water molecules via infrared resonance, accelerating enzymatic hydrolysis and substrate accessibility [18,38,39]. These combined effects align with previous findings showing improved growth and feed utilization in fishes fed dietary SiNPs [18,40]. Although the BV and BVCS treatments alone did not significantly increase the SGR or RGR, the inclusion of SiNPs in BVSiNPCS restored these improvements, highlighting the pivotal role of SiNPs in optimizing the growth-promoting effects of *B. velezensis* and chitosan [19,41].

Serum biochemical analysis revealed no significant alterations in most parameters across dietary treatments, indicating that the functional additives used did not induce systemic toxicity or disrupt metabolic homeostasis. However, compared with those of the other groups, the serum calcium concentrations of the fish fed the BVSiNPCS diet were significantly lower. While direct mechanistic evidence was not isolated in this study, we hypothesize that this reduction involves the ion-binding properties of the additives. Silica nanoparticles bind calcium ions (Ca^2+^) via their negatively charged surface groups, such as silanol (Si–OH), facilitating electrostatic interactions [42]. Chitosan binds Ca^2+^ through noncovalent, electrostatic interactions involving its amino (–NH_2_), hydroxyl (–OH), and acetylamino (–NHCOCH_3_) groups [43,44]. Together, these materials may synergistically enhance ion adsorption by offering a broader array of binding sites and complementary surface chemistries. This could lead to (1) the redistribution of calcium within the body [43,44] or (2) the microbial utilization of surface-bound ions by diverse gut microbiota. Importantly, the observed decrease in serum calcium does not necessarily reflect systemic deficiency, as baseline calcium levels in fish vary widely (2.51–16 mg/dL) [45,46].

In contrast, amylase activity was significantly lower in the BVSiNP group than in the other treatment groups, despite the maintenance of growth performance. This suggests a reduced physiological demand for endogenous enzyme secretion due to microbial enzymatic compensation. The physicochemical properties of SiNPs may increase nutrient bioavailability [18,38,39], whereas the increased relative abundance of *Cetobacterium somerae*, a carbohydrate-metabolizing taxon [24,47], contributes to gut fermentation. The synergistic effects among SiNPs, *C. somerae*, and extracellular carbohydrases secreted by *B. velezensis* [48] likely promoted more efficient carbohydrate digestion and assimilation, thereby reducing the host’s reliance on endogenous digestive enzymes. These metabolic interactions are corroborated by functional annotation of the gut microbiota, which revealed enrichment in COG categories associated with amino acid, nucleotide, and carbohydrate metabolism.

All supplemented groups presented significantly elevated respiratory burst activity, with the strongest responses in the BVCS and BVSiNPCS groups. This activation is likely driven by probiotic-associated microbial stimuli [49], nanoparticle-induced metabolic stimulation [50], and TLR-mediated signaling triggered by chitosan [51]. These findings align with previous reports that *Bacillus* probiotics increase respiratory bursts [49], whereas chitosan augments innate immunity by stimulating superoxide and nitric oxide production, neutrophil activity, and phagocyte activation [52]. The enhanced oxidative response likely reflects synergistic immunostimulation via microbial pattern recognition, nanoparticle-induced metabolic activation, and chitosan-mediated TLR signaling.

Significant modulation of key innate immune genes was observed. The expression of *α2M* was distinct across tissues, indicating localized and formulation-specific immune modulation. In the gills, markedly high *α2M* expression was observed in the BVCS and BVSiNPCS groups, followed by the BVSiNP group. Given that the gills serve as the first line of defense and that *α2M* functions as a broad-spectrum protease inhibitor, these findings support the role of synbiotic chitosan and SiNPs in enhancing gill mucosal immunity against external pathogens [53,54]. Similarly, the expression of heat shock protein 70 (*HSP70*), a molecular chaperone involved in cellular protection [55], increased in response to probiotic SiNP-supplemented diets (BVSiNP) in the gills and liver. This finding aligns with findings in other fish species in which *HSP70* upregulation was associated with improved stress resistance following probiotic treatment [56,57]. Additionally, the expression of *Mx*, an interferon-induced gene with critical antiviral and antibacterial functions [58,59], was markedly upregulated in the gills and liver of fish fed probiotic SiNP-containing diets. Complement component 3 (*C3*), a pivotal effector of the complement system [60], was also significantly elevated in the livers of the BVSiNP group. Given that *C3* is synthesized primarily in hepatic tissues [61], this upregulation suggests that systemic humoral immune activation contributes to the improved disease resistance observed in BVSiNP-treated fish.

Gut microbiota profiling revealed substantial shifts in microbial composition. Fish fed BV, BVSiNP, and BVCS diets exhibited a marked dominance of *Cetobacterium somerae*, a beneficial anaerobe known for vitamin B_12_ synthesis [24,62], whereas the control group was enriched in Proteobacteria, including opportunistic genera such as *Vibrio* and *Photobacterium* [63]. The enrichment of *C. somerae* may be attributed to the antimicrobial activity of *B. velezensis*, which produces compounds that suppress opportunistic pathogens, thereby reducing microbial competition [64,65]. Moreover, *C. somerae* is a key producer of vitamin B_12,_ which plays essential roles in host lipid metabolism and gut barrier integrity [24,64,65]. Despite its dietary inclusion, *B. velezensis* was not detected in the gut microbiota, suggesting a transient presence with indirect ecological influence via redox modulation that favors anaerobes such as *C. somerae* [24,66].

The functional classification of the gut microbiota based on COG categories offers important insights, although importantly, these are inferred metabolic potentials rather than validated enzymatic activities. In the present study, metabolism-related COGs—including those involved in amino acid, nucleotide, and carbohydrate metabolism—were significantly enriched in the probiotic-treated groups. These findings support the hypothesis of enhanced microbial nutrient biosynthesis and host assimilation processes [22,23,65]. These genes likely contribute to the microbial production of essential amino acids, B vitamins, and SCFAs, which play critical roles in regulating intestinal energy metabolism and feed efficiency [24,66,67]. The enrichment of defense mechanism-associated COGs supports the strong establishment of *C. somerae* in the BV, BVSiNP, and BVCS groups, indicating that *C. somerae*-dominated guts possess enhanced protective functions that may suppress the proliferation of opportunistic pathogens [24,68].

Histological analysis further corroborated the benefits of the nanoparticles. The increased villus height and goblet cell density observed in the BVSiNPCS group indicate that probiotics, SiNPs, and chitosan synergistically support mucosal integrity. Taller villi increase the absorptive surface area, thereby improving nutrient uptake efficiency [69], whereas higher goblet cell density strengthens the mucus barrier against pathogenic intrusion [70]. The complementary effects of SiNPs, which increase enzymatic activity and mitigate oxidative stress [71], and chitosan, which stimulates mucin secretion and maintains epithelial integrity [72], likely explain the superior intestinal morphology in the combined treatment group. The strong positive correlation between villus height and goblet cell density underscores the interdependence of absorptive and protective intestinal functions.

Despite these structural improvements in the gut mucosa, postchallenge survival analysis revealed a divergence in outcomes. Dietary supplementation with BV and BVSiNP markedly increased the resistance of Asian seabass to *V. vulnificus*, as evidenced by the significantly higher survival rates of these groups than those of the control. The increased survival in the BV and BVSiNP groups was accompanied by the upregulation of key innate immune markers (*α2M*, *HSP70*, *Mx*, and *C3*) and a gut microbiota structure dominated by *C. somerae*. These findings support the hypothesis that immune potentiation and microbiota-mediated protection jointly contribute to disease resistance [23,47,49]. However, in contrast to the improved gut morphology and immune gene expression, the triple combination group (BVSiNPCS) exhibited only partial protection. This presents a complex paradox. The reduced abundance of *C. somerae* in this group suggests that the absence of dominant beneficial taxa may compromise microbiota functionality [23,73,74]. Furthermore, the discrepancy suggests that excessive immune stimulation (or “immunostimulatory fatigue”) combined with the aforementioned reduction in serum calcium bioavailability may have compromised the overall physiological resilience of the fish in this group [45,46,75]. Therefore, while the BVSiNP formulation clearly provides synergistic benefits for both growth and survival, the triple combination requires further optimization to balance immune activation with homeostatic maintenance.

## 5. Conclusions

This study demonstrated that dietary supplementation with *B. velezensis* AAHM-BV2301, chitosan, and SiNPs modulates host physiology, the gut microbiota, and immune responses in Asian seabass fingerlings. The BVSiNP formulation most effectively improved growth, immune activation, and resistance to *V. vulnificus*, likely by acting as a physicochemical nanoscaffold that enhances probiotic bioavailability. While the BVSiNPCS diet promoted the greatest increase in microbial diversity and network complexity, its protective efficacy was compromised, suggesting a trade-off between microbial richness and physiological homeostasis. These findings highlight the potential of nanoparticle-based delivery systems for microbiome-targeted health management in aquaculture.

## Data Availability

The data that support the findings of this study are available on request from the corresponding authors.

## Supplementary Materials

The following supporting information can be downloaded at: https://www.mdpi.com/article/10.3390/biom16010088/s1, Table S1: Sequences of primers used for qRT–PCR analysis of immune-related genes in Asian seabass. Table S2: High-throughput sequencing of the *16S rRNA* gene generated a total of 713,965 raw circular consensus sequences (CCSs) across all the samples. Figure S1: Rarefaction curves for all the samples of the majority of the microbial diversity.
