# Supplementary material for: Synergistic Effects of Silica Nanoparticles, Chitosan and Bacillus velezensis AAHM-BV2301 on the Growth, Immunity, Gut Microbiota and Disease Resistance of Asian Seabass (Lates calcarifer)
Source: Biomolecules. 2026 Jan 5;16(1):88. doi: 10.3390/biom16010088 (PMC12838700; doi:10.3390/biom16010088)

Supplementary Materials Table S1, Tangal et al. (2025)

Gene	Primer name	Nucleotide sequences (5′ → 3′)	Amplicon size (bp)	Tm (°C)	Refences
**Hepcidin-1 (*Hep1*)**	Lc_Hep1	F: ATTTGCATTCTGCGAAGCTCTGCC	208	60	[9]
		R: CCATTGACATCTCTTGATGTGCCG			
**α-2-macroglobulin (*a2M*)**	Lc_A2M	F: TGCTTTCCTGGTTTTGTCC	136	60	[9]
		R: TGGTTGATGCCTATGTGCC			
**Complement 3 (*C3*)**	Lc_C3	F: GCAATCCTCCACAACTACAG	111	60	[9]
		R: ACTCTGCCTCCTGACAGATAC			
**CC chemokine (*CC*)**	Lc_CC	F: CCTGCCCTGTGCTCAATGC	111	60	[9]
		R: TTGCCTGGGCTTCTTGTTAG			
**Lysozyme (*Lyz*)**	Lc_Lyz	F: TGCATCACACACAGGTGTCAG	401	60	[9]
		R: CATCCAGCTCATAGTAGTCAGC			
**Heat shock protein 70kDa (*HSP70*)**	Lc_HSP70	F: AAGGCAGGAGGTGATGTGTC	186	60	[9]
		R: TGGCCTCGTTCTTCTTCTTC			
**Interleukin-8 (*IL-8*)**	Lc_IL-8	F: TGATCCTGCGATGCTCGATGCAT	206	60	[9]
		R: AGGGTCTGTCGTCAGCTGTGTTC			
**Myxovirus resistance gene (*Mx*)**	Lc_Mx	F: GAGGTCATCCACCTGAAGAAGG	184	60	[9]
		R: GAGGTCATCCACCTGAAGAAGG			
**β-actin (*β-actin*)**	Lc_β-actin	F: CTTTACCACACCAGCAGATGTGG	157	60	[9]
		R: TGGCCGAGGACTTTGATGTA			
**18S ribosomal RNA (*18srRNA*)**	Lc_18srRNA	F: AACGAGACTCCGGCATGCTA	103	60	[31]
		R: CCGGACATCTAAGGGCATCA			

Supplementary Materials Table S2, Tangal et al. (2025)

Sample ID	Raw CCS	Clean CCS	Effective CCS	Avg. Lenght (bp)	Effective (%)
**C1**	40,314	40,310	40,278	1,474	99.91
**C2**	35,007	35,007	34,945	1,474	99.82
**C3**	34,830	34,826	34,757	1,457	99.79
**C4**	31,238	31,231	31,203	1,453	99.89
**BV1**	30,439	30,434	30,345	1,440	99.69
**BV2**	35,272	35,260	35,041	1,441	99.35
**BV3**	40,861	40,855	40,644	1,438	99.47
**BV4**	33,861	33,857	33,648	1,438	99.37
**BVCS1**	40,104	40,104	40,048	1,442	99.86
**BVCS2**	31,138	31,128	31,022	1,443	99.63
**BVCS3**	36,664	36,663	36,642	1,435	99.94
**BVCS4**	36,744	36,741	36,721	1,436	99.94
**BVSiNP1**	37,279	37,279	37,260	1,435	99.95
**BVSiNP2**	35,680	35,675	35,633	1,435	99.87
**BVSiNP3**	36,010	36,010	35,956	1,434	99.85
**BVSiNP4**	33,959	33,945	33,882	1,434	99.77
**BVSiNPCS1**	36,882	36,863	35,930	1,462	97.42
**BVSiNPCS2**	35,577	35,554	35,357	1,462	99.38
**BVSiNPCS3**	36,165	36,144	35,904	1,463	99.28
**BVSiNPCS4**	35,941	35,938	35,882	1,468	99.84

Supplementary Materials Figure S1, Tangal et al. (2025)

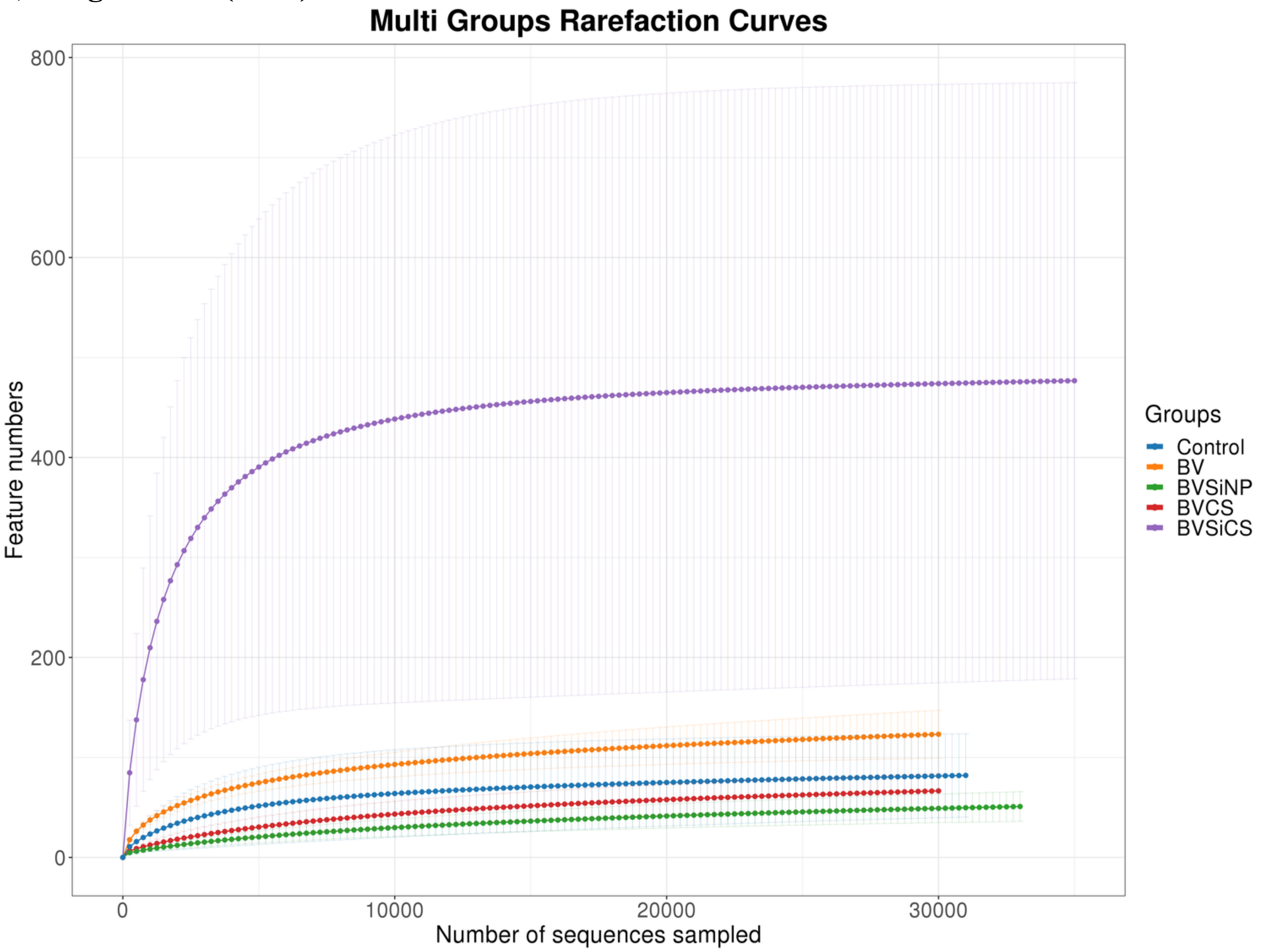

Supplement: Supplementary file 1 [file biomolecules-16-00088-s001.zip › biomolecules-4009407-supplementary.pdf]
